# HGUE-C-1 is an atypical and novel colon carcinoma cell line

**DOI:** 10.1186/s12885-015-1183-3

**Published:** 2015-04-08

**Authors:** Silvina Grasso, Isabel Martínez-Lacaci, Víctor Manuel Barberá, Adela Castillejo, José Luis Soto, Javier Gallego-Plazas, Natividad López-Riquelme, Pilar García-Morales, Trinidad Mata-Balaguer, José Antonio Ferragut, Miguel Saceda

**Affiliations:** 1 Instituto de Biología Molecular y Celular, Universidad Miguel Hernández, 03202 Elche, Alicante, Spain; 2 Unidad AECC de Investigación Traslacional en Cáncer, Hospital Clínico Universitario Virgen de la Arrixaca, 30120 Murcia, Spain; 3 Unidad de Investigación, Hospital General Universitario de Elche, 03203 Elche, Alicante, Spain; 4 Servicio de Análisis Clínicos, Hospital Clínico Universitario Virgen de la Arrixaca, 30120 Murcia, Spain; 5 Fundación para el Fomento de la Investigación Sanitaria y Biomédica de la Comunidad Valenciana (FISABIO), Hospital General Universitario de Elche, 03203 Elche, Alicante, Spain; 6 Instituto de Neurociencias, Universidad Miguel Hernández de Elche, 03550 San Juan, Alicante, Spain

**Keywords:** Colon carcinoma, Cell line, Chemoresistance

## Abstract

**Background:**

Colorectal carcinoma is a common cause of cancer. Adjuvant treatments include: 5-fluorouracil administered together with folinic acid, or more recently, oral fluoropyrimidines such as capecitabine, in combination with oxaliplatin or irinotecan. Metastatic colorectal cancer patients can benefit from other additional treatments such as cetuximab or bevacizumab.

**Methods:**

Using cell culture techniques, we isolated clonal populations from primary cultures of ascitic effusion derived from a colon cancer patient and after several passages an established cell line, HGUE-C-1, was obtained. Genetic analysis of HGUE-C-1 cells was performed by PCR of selected exons and sequencing. Cell proliferation studies were performed by MTT assays and cell cycle analyses were performed by flow cytometry. Retinoblastoma activity was measured by luciferase assays and proteins levels and activity were analysed by Western blot or immunohistochemistry.

**Results:**

We have established a new cell line from ascitic efussion of a colon cancer patient who did not respond to 5-fluorouracil or irinotecan. HGUE-C-1 cells did not show microsatellite instability and did not harbour mutations in *KRAS*, *BRAF*, *PI3KCA* or *TP53*. However, these cells showed loss of heterozygosity affecting Adenomatous Polyposis Coli and nuclear staining of β-catenin protein. The HGUE-C-1 cell line was sensitive to erlotinib, gefitinib, NVP-BEZ235, rapamycin and trichostatin, among other drugs, but partially resistant to heat shock protein inhibitors and highly resistant to AZD-6244 and oxaliplatin, even though the patient from which this cell line was derived had not been exposed to these drugs. Molecular characterization of this cell line revealed low expression levels and activity of Retinoblastoma protein and elevated basal levels of Erk1/2 activity and p70S6K expression and activity, which may be related to chemoresistance mechanisms.

**Conclusions:**

HGUE-C-1 represents a novel and peculiar colon carcinoma model to study chemoresistance to chemotherapeutic agents and to novel anti-neoplasic drugs that interrupt signalling pathways such as the APC/βcatenin, Ras/Raf/Mek/Erk, PI3K/mTOR/p70S6K pathways as well as histone regulation mechanisms.

## Background

Colorectal cancer (CRC) is the third most common type of cancer, constituting approximately 9.4% of all cancers diagnosed, and the third leading cause of cancer death. Approximately, 25% of patients diagnosed of colorectal cancer have already metastatic spread, and 25% of initially localized disease will develop metastases [1]. The recommended strategy for locally advanced colon cancer consists of preoperative combined chemo and radiotherapy, followed by surgery and then by adjuvant chemotherapy, to reduce both the local recurrence and the possibility of distant relapse [2]. Adjuvant chemotherapy in stage III colon cancer is fully justified. The combination of 5-fluorouracil (5-FU) and folinic acid (leucovorin), which enhances 5-FU effects by inhibiting thymilydate synthase, administered for six months, or oral fluoropyrimidines such as the 5-FU prodrugs capecitabine [3] or tegafur [4], are able to reduce the risk of death by 30%, assuming an absolute increase of 10-13% in terms of survival. Further combination of oxaliplatin either to the regimen of 5-FU and folinic acid or to capecitabine increased survival in this group of patients [5-7]. Indication and treatment of stage II disease is controversial and depends on clinical and histological risk factors [8]. Considering advanced disease, first-line chemotherapy including the combination of folinic acid with continuous infusion of 5-FU, along with oxaliplatin (FOLFOX) or irinotecan (FOLFIRI) are reasonable choices, with comparable effectiveness in terms of objective responses and survival [9]. Capecitabine combinations have shown similar efficacy to continuous infusion of 5-FU combinations [10-13]. Bevacizumab, a humanized monoclonal antibody against vascular endothelial growth factor (VEGF), and cetuximab, a monoclonal antibody directed against the extracellular domain of the epidermal growth factor receptor (EGFR), have demonstrated an additive effect on chemotherapy, therefore broadening the options for treatments of patients in first and consecutive lines [14,15]. However, anti-EGFR therapy is only recommended in KRAS/BRAF wild type patients.

Recently, we have established primary cultures obtained from patients with CRC cancer. The novel colon cancer cell line HGUE-C-1 was obtained from ascitic efussion of a 76-year old man with colon cancer. The patient was initially admitted at the hospital, in November 2003. A complete colonoscopy was performed, showing a mass eight centimetres long, starting eighteen centimetres far from the anal verge. The biopsy confirmed diagnosis of colon adenocarcinoma. R0 down anterior resection was performed, resulting in a moderately differentiated colon adenocarcinoma affecting serosa, with two out of eleven metastatic lymph nodes (GII pT3 N1b). After postoperative evaluation consisting of thorax-abdomen-pelvic CT scan which showed no evidence of residual disease, and carcinoembrionary antigen (CEA) value in the normal range, adjuvant chemotherapy with 5-FU and folinic acid was administered for six months. After completing adjuvant chemotherapy, there was no suspect or evidence of relapse by imaging tests and blood analysis. The patient entered at that time onto a scheduled follow-up programme. Two years later, the patient was admitted into the hospital due to an increase in the abdominal perimeter. Thorax-abdomen-pelvic CT scan revealed massive ascites. The cytology performed on ascitic fluid obtained after diagnostic paracentesis confirmed colon adenocarcinoma origin of the ascites. Palliative chemotherapy was then started with capecitabine and irinotecan. After a second cycle of chemotherapy, the patient was admitted into the hospital due to grade III chemotherapy-induced diarrhea. During admission, two parecenteses of malignant haematic ascites with 72 hours interval were performed (5,000 and 5,400 ml). The patient received a third cycle of treatment on an outpatient basis with 25% reduction in doses of capecitabine and irinotecan. Seven days after beginning of the third cycle, the patient was again admitted into the hospital due to grade III diarrhea associated to renal failure. The patient showed no response to medical procedures, and considering the global prognosis, exclusive symptomatic treatment was decided. Patient died eight days after final admission.

The new HGUE-C-1 cell line constitutes an interesting model of study from several points of view. Initially, we determined whether HGUE-C-1 cells presented the microsatellite instability (MSI) phenotype, already known to be related to colorectal carcinogenesis in about 15% of all CRC [16]. Interestingly, HGUE-C-1 cells did not show MSI phenotype. HGUE-C-1 cells were also analysed for mutations in *KRAS*, *BRAF*, *PIK3CA* and *TP53* genes, which are quite commonly mutated in colon carcinoma and have been related to colon carcinogenesis [17-19]. Further analysis with the dinucleotide polymorphic repeat marker D5S346 showed loss of heterozygosity affecting the Adenomatous Polyposis Coli (APC) containing region in chromosome five and nuclear staining of β-catenin protein, suggesting that the APC signalling pathway was modified in HGUE-C-1 cells.

HGUE-C-1 cells are also interesting as an experimental model for the study of chemoresistance in patients with colon cancer. In this sense, HGUE-C-1 cells show resistance to 5-FU and irinotecan. This cell line constitutes a better physiological model for chemoresistance studies in comparison with other cell lines that become resistant in vitro by selective pressure after treatment with increasing concentrations of specific drugs.

HGUE-C-1 represents an established cell line derived from primary cultures of a biological sample obtained from a patient, in the context of a general project aimed to the development of predictive tests with a panel of different alternative treatments. In this context, a complete pharmacological profile of HGUE-C-1 cells was performed. Interestingly, the HGUE-C-1 cell line showed chemosensitivity to EGFR inhibitors erlotinib, gefitinib, the dual PI3K/mTOR inhibitor NVP-BEZ235, the mTOR inhibitor rapamycin, the histone deacetylase inhibitor trichostatin (TSA) among other drugs, being partially resistant to the heat shock protein 90 (Hsp90) inhibitor 17-allylamino-17-demethoxygeldanamycin (17-AAG), and totally resistant to the Mek inhibitor AZD-6244 (Selumetinib) and to the chemotherapeutic agent oxaliplatin, despite that the patient was not treated with such drugs. The putative molecular mechanisms involved in HGUE-C-1 carcinogenesis, and drug chemosensitivity or chemoresistance will be discussed herein.

## Methods

### Cell culture

The human colorectal cancer cell lines HT-29, SW620, SW480, HCT-15 and HCT-116 cells were obtained from the Instituto Municipal de Investigaciones Médicas de Barcelona (Spain), HT-29, SW480, HCT-15, HGUE-C-1, SW620 and HCT-116 cells were maintained in Dulbecco’s modified Eagle’s medium (DMEM) (Labclinics SA, Barcelona, Spain) supplemented with 10% heat-inactivated fetal bovine serum (FBS) (Labclinics), 50 U/mL of penicillin and 50 mg/mL streptomycin (Labclinics) and incubated at 37°C in a humidified 5% CO_2_/air atmosphere.

### Reagents

Gefitinib, erlotinib, sorafenib, 17-AAG, NVP-BEZ235 and AZD-6244 were obtained from ChemieTek (Indianapolis, IN, USA). Rapamycin, tricostatin (TSA), propidium iodide and 3-(4, 5-Dimethylthiazol-2-yl)-2,5-diphenyltetrazolium bromide (MTT) were purchased from Sigma-Aldrich (St. Louis, MO, USA). RNase A was obtained from Serva (Heidelberg, Germany).

### Cell proliferation assays

Cell proliferation was assessed using the MTT assay based on the activity of the mitochondrial enzyme succinate dehydrogenase. Colorectal carcinoma cells were seeded in 96-well plates at a density of 2,500 cells per well and incubated at 37°C with 5% CO_2_. Increasing doses of the indicated drugs were added, with DMSO as non-treated control. The dose range for each drug was selected taking in consideration the maximal and the minimal concentration of the drug in patient´s plasma and/or previous MTT assays dose response studies in our panel of colon cancer cell lines. The culture was continued for 72 hours and at the end of the treatment, 30 μl of MTT solution (5 mg/ml in PBS) were added into each well, followed by incubation at 37°C for three hours. The culture medium containing MTT was aspirated and the formazan crystals formed were then solubilized with 200 μl DMSO for 30 minutes. Absorbance was measured at wavelength 570 nm in a microplate reader (Anthos 2001 Labtec Instruments GmbH, Wals, Austria), and the percentage of proliferation of HGUE-C-1 and HT-29 cells was determined for each concentration of the indicated drug. Both treatment and control groups were performed in 6 replicate wells and the experiment was repeated at least three times to ensure the data reproducibility.

### Cell cycle analysis

Cells were cultured in T25 flasks and treated with the different drugs at the indicated concentration with DMSO as non-treated control. At the end of the treatment, both floating and adherent cells were collected, combined, washed with PBS, and fixed with 500 μl of chilled 70% ethanol in PBS at −20°C. Following fixation, cells were pelleted and resuspended in 500 μl of PBS containing 0.5% Triton X-100, 25 μg/ml RNase A and 25 × 10^−3^ μg/ml propidium iodide. After 30 minutes at room temperature in the dark, cell cycle distribution was determined with an Epics XL flow cytometer (Beckman Coulter Co., Miami, FL). When the fluorescence is less than the fluorescence peak corresponding to that of the G_0_/G_1_-phase, cells are considered to be apoptotic (sub-G_1_).

### Western blot analysis

Cells were plated for different times in serum-starved conditions, lysed in NP-40 lysis buffer (50 mM Tris–HCl pH 7.4, 1% NP-40, 150 mM NaCl, 5 mM EDTA, 50 mM NaF, 30 mM Na_4_P_2_O_7_, 1 mM Na_3_VO_4_,) with a protease inhibitor cocktail (Sigma-Aldrich) for 30 min on ice. After centrifugation at 15,000 x g for 5 minutes at 4°C, the supernatants were collected and the protein concentration of the cell lysates was determined by the Bradford method (Bio-Rad, Richmond, CA). Total protein content (60–80 μg) from each lysate was electrophoresed on 10% polyacrylamide gels in the presence of sodiumdodecyl sulfate (SDS) and transferred onto nitrocellulose membranes. Nonspecific sites were blocked incubating for 1 hour at room temperature with 5% non-fat dry milk. Membranes were then incubated overnight with the indicated primary antibodies. EGFR and HER3 antibodies were purchased from Santa Cruz Biotechnology (Santa Cruz, CA, USA); phospho-ERK1/2, ERK1/2, phospho-p70S6K, p70S6K, phospho-RPS6 and RPS6 antibodies were purchased from Cell Signaling Technologies (Danvers, MA); Retinoblastoma antibody was purchased from BD Biosciences (San Jose, CA); actin antibody was purchased from Sigma-Aldrich. The primary antibodies were incubated with secondary antibodies linked to horseradish peroxidase (GE Healthcare, Buckinghamshire, UK). Specific proteins were visualized by enhanced chemiluminescence detection using the ECL system (GE Healthcare), according to the manufacturer’s instructions.

### Transfection and luciferase measurement

HT-29 and HGUE-C-1 cells were seeded in 24-well culture plates (180,000 and 150,000 cells/well, respectively). When cells reached 40–60% confluence (24 hr), they were transiently transfected with 0.8 μg of pRb-TA-Luc or pE2F-TA-Luc responsive luciferase reporter plasmid (Clontech, Mountain View, CA), using Lipofectamine2000 reagent (Invitrogen, Carlsbad, CA, USA) in a DNA transfection agent ratio of 1:3. The pTa-Luc vector lacking the response element was used as negative control. Six hours after transfection, culture medium was replaced by fresh medium and cells were kept under normal growth condition in DMEM with 10% foetal serum for 24 hours prior to lysis and luciferase detection. All transfections were performed as co-transfections using a Renilla luciferase expression plasmid (Promega Corporation, Madison, WI) to establish an internal control for transfection efficiency. The transfected cells were washed once with PBS and lysed using 100 μl Passive Lysis Buffer (Promega). Firefly and Renilla luciferase activities were determined using the Dual-Luciferase Reporter Assay System (Promega) according to the manufacturer’s instructions, using for detection of the chemiluminescent signal, a luminometer from Berthold (Montreal Biotech, Kirkland, QC). Promoter activities of the RB and E2F reporter plasmids were expressed using the arbitrary units “RLU” (relative luciferase units).

### HGUE-C-1 genetic analysis

DNA from different passages of HGUE-C-1 cells was isolated using the DNeasy Blood & Tissue Kit (Qiagen, Valencia, CA). HGUE-C-1 cells were screened for MSI status by multiplex PCR using the pentaplex set of monomorphic markers (BAT 26, BAT 25, NR21, NR24 and NR27). Mutations in *BRAF* (V600E) and *KRAS* (codon 12/13) were identified by direct sequencing. Primer sequences were BRAF:F-TGCTTGCTCTGATAGGAAAATGA, BRAF:R-TGGATCCAGACAACTGTTCAAA, KRAS.F-GCCTGCTGAAAATGACTGAA and KRAS:R- AGAATGGTCCTGCACCAGTAA. PCR products were purified using a QIAquick PCR purification kit (Qiagen) and directly sequenced on an ABI PRISM 3100 sequencer (Applied Biosystems, Foster City, CA). Presence of *PIK3CA* mutations in exons 9 and 20 were determined by direct sequencing after a nested PCR reaction. Amplicons were purified and subjected to direct sequencing using exosap-kit (USB Corporation) and sequenced using the primers PI542-5IF and PI542-5IR for exon 9 and PI1047IF and PI1047IR for exon 20 with BigDye Terminator Sequencing Kit and ABI PRISM 3100 sequencer (Applied Biosystems). *TP53* gene mutations were determined on genomic DNA from two different culture passages, p3 and p30, of HGUE-C-1 cells. Mutations in exons 4–11 were analysed by PCR amplification and direct sequencing of the coding sequence and exon-intron boundaries. The PCR amplified products were purified and sequenced. Three dinucleotide polymorphic repeat markers (D2S123, D5S346 and D17S250) were amplified by PCR using fluorescent labelled primers and analysed by capillary electrophoresis in an ABI PRISM 3100 sequencer (Applied Biosystems). The size of the markers was compared at two different cell culture passages p3 and p45.

### Immunohistochemistry

Three**-**micrometre sections were cut from paraffin-embeded tumour specimens and the slides were pretreated in a PT link from Dako (Glostrup, Denmark) and incubated with antibodies against β-catenin. Signal amplification and development were carried out with EnVision Flex kit (Dako), according to the manufacturer’s instructions.

### Ethics statement

Ascitic efussion (from where HGUE-C-1 cells were derived) of a patient with colon cancer was obtained according to institutional guidelines with the approval of the “Comité Etico y de Investigación Clínica” (Clinical Research and Ethics Committee) Review Board, Hospital General Universitario de Elche, Elche, Spain. This ethics committee waived the need for informed consent for this particular kind of human samples.

## Results

### Phenotypical characterization of the HGUE-C-1 cell line

**Figure 1:**
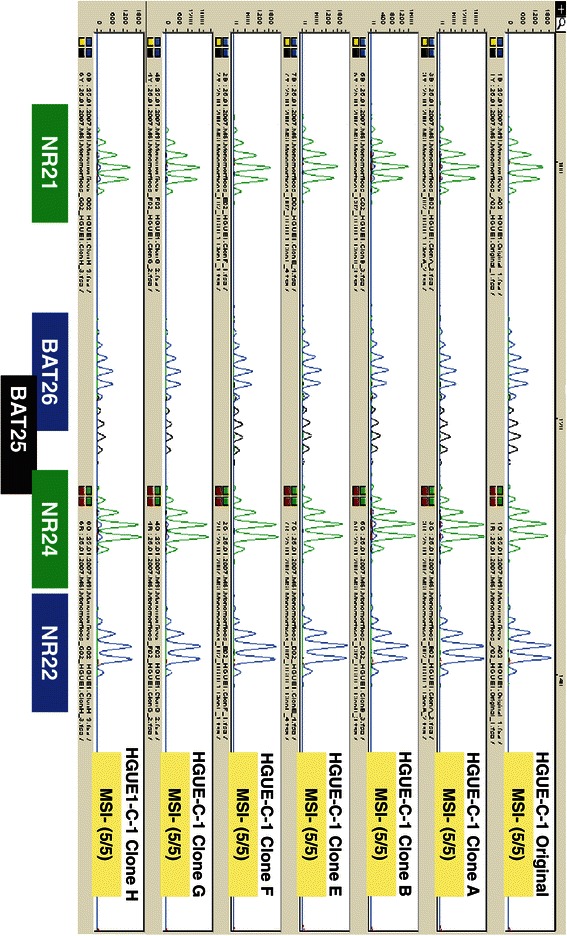
**Phenotypical characterization of the HGUE-C-1 cells.** Probes for the pentaplex set of monomorphic markers were used to determine MSI on DNA samples from passage 3 of HGUE-C1 cells and from passage 15 of clones A, B, E, F, G and H.

Initially, the DNAs obtained from early passages of HGUE-C-1 cells (passage 3) and from the individual clones of HGUE-C-1 (A,B,E, F,G and H) isolated after passage 15 of the original HGUE-C-1 culture, were analysed for microsatellite instability (MSI) status with probes for the pentaplex set of monomorphic markers (NR21, BAT25, BAT26, NR22 and NR24). Results in Figure 1 demonstrate that the parental HGUE-C-1 cells as well as the HGUE-C-1 clonal populations A, B, E, F, G and H do not present MSI phenotype. Next, DNA from early and late passages of HGUE-C-1 cells was evaluated for presence of mutations on *TP53*, *BRAF* (V600E), *KRAS* (codons 12 and 13) and *PIK3CA* (codons 9 and 20). Results in Table 1 indicate that different passages of the HGUE-C-1 cell line do not harbour mutations in any of the analysed genes.

**Table 1:** **DNA from early and late passages of HGUE-C-1 cells was tested for mutational status of** ***TP53*** **,** ***BRAF*** **(V600E),** ***KRAS*** **(codons 12 and 13) and** ***PIK3CA*** **(exons 9 and 20)**

	Passage 3	Passage 36
TP53	wt	wt
BRAF	wt	wt
KRAS	wt	wt
PIK3CA	wt	wt

**Figure 2:**
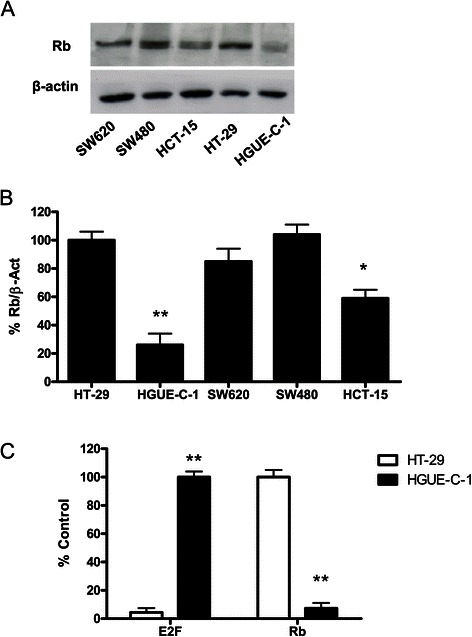
**Retinoblastoma (Rb) protein expression and activity.** HGUE-C-1 and HT-29, SW620, SW480 and HCT-15 colorectal carcinoma cells were grown, lysed and subjected to Western blot analyses to detect levels of Rb or β-actin, using specific antibodies. β-actin was used as a loading control **(2A)**. Densitometric values of Rb bands were normalized to β-actin bands and represented as the average of at least three experiments. Error bars are the S.E.M. **(2B)**. Retinoblastoma activity was determined on HT-29 and HGUE-C-1 cell lines by transient transfection assays with Rb and E2F reporter genes and represented as the average of at least four experiments. Error bars are the S.E.M. **(2C)**.

**Figure 3:**
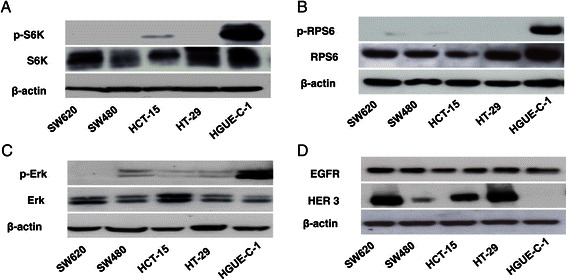
**Expression and activity of signalling molecules.** HGUE-C1, HT-29, SW620, SW480 and HCT-15 cells were grown, serum-starved for 48 hours, lysed and subjected to Western blot analyses using antibodies against p70S6K and phosphorylated p70S6K (p-pS6K). The p85S6K isoform is also shown **(3A)**. RPS6 and phosphorylated RPS6 (p-RPS6) levels were also determined by Western blot in these cell lines after serum starvation using specific antibodies **(3B)**. Erk 1/2 and phosphorylated Erk1/2 (p-Erk 1/2) levels were also determined in these cell lines after serum starvation by Western blot **(3C)**. HGUE-C-1, HT-29, SW620, SW480 and HCT-15 cells were grown, lysed and subjected to Western blot analyses using antibodies against EGFR and HER3 **(3D)**.

Western blot analysis of different proteins involved in signal transduction pathways were performed in order to find differences between HGUE-C-1 cells and others well characterized colon carcinoma cell lines, such as HT-29. As shown in Figure 2A and B, Retinoblastoma (Rb) protein levels are lower in HGUE-C-1 cells when compared with HT-29, SW620, SW480 and HCT-15 cells. Rb activity, determined by transient transfection assays with Rb and E2F reporter genes, is also lower in HGUE-C-1 cells, as shown in Figure 2C. HGUE-C-1 cells show high expression levels of p70S6K (RPS6KB1) protein under serum-starved conditions, as compared with HT-29, SW620, SW480 and HCT-15 cells (Figure 3A). In addition, p70S6K is constitutively active in HGUE-C-1 cells, as demonstrated by high levels of phosphorylated p70S6K (Figure 3A) and by the presence of phosphorylated RPS6, its downstream target (Figure 3B). Likewise, Erk1 and Erk2 appear constitutively active (Figure 3C) under serum-starved conditions in HGUE-C-1 cells, in contrast to the activity level of these proteins observed in HT-29, SW620, SW480 and HCT-15 cells. However, when Akt, RSK and other key protein were analysed no significant differences in activity and levels were observed between both cell lines (data not shown). Finally, protein levels of members of the HER receptor family such as EGFR and HER3 were analysed in HGUE-C-1 and others colon cancer cell lines. HGUE-C-1 cells do not express HER3 protein, whereas HT-29, SW620, SW480 and HCT-15 cells express this receptor (Figure 3D).

To determine whether APC gene was affected in HGUE-C-1 cells, the dinucleotide polymorphic repeat marker D5S346 was studied in DNA samples obtained from two different passages of HGUE-C-1 cells, as well as in DNA obtained from non- tumour cells from the same patient. HGUE-C-1 cells showed loss of heterozygosity in APC related region of chromosome 5 (data not shown).

### Pharmacological profile of HGUE-C-1 cells

**Figure 4:**
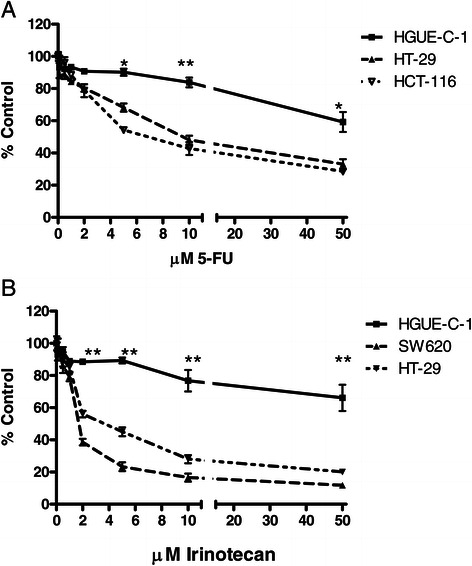
**Effects of 5-FU (4A) and irinotecan (4B) on HGUE-C 1, HT-29, HCT-116 and SW620 cell proliferation.** Sensitivity to both antineoplasic agents was also determined in HGUE-C-1, HT-29, HCT-116 and SW620 cells by MTT assays. Cells were grown in 96-well plates, treated with different concentration of drugs for 72 hours and incubated with MTT. Proliferation rates were determined by colourimetry and the average of at least four experiments is represented and referred as percentage of control. Error bars are the S.E.M. *p < 0.05, **p < 0.01 versus HT-29 values.

**Figure 5:**
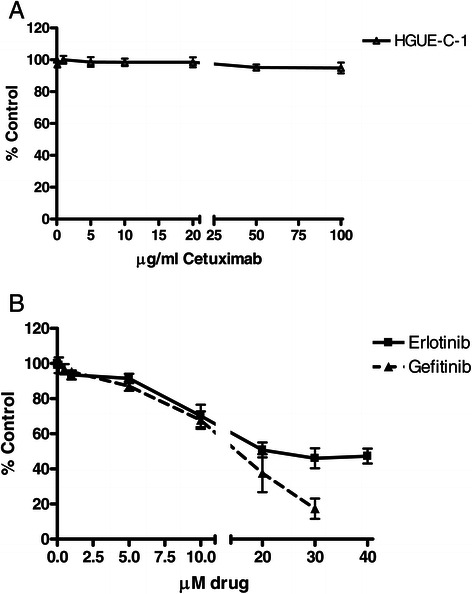
**Effects of the EGFR inhibitors on cell proliferation.** The effects of cetuximab **(5A)**, erlotinib **(5B)**, and gefitinib **(5B)** on proliferation of HGUE-C-1 cells were determined by MTT assays. The average of at least four experiments is represented and referred as percentage of control. Error bars are the S.E.M.

**Figure 6:**
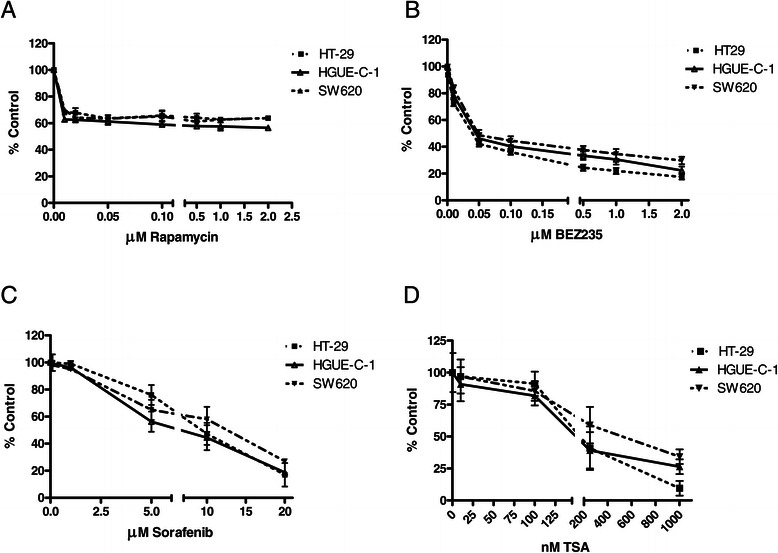
**Effects of different drugs on cell proliferation.** The effects of rapamycin **(6A)**, NVP-BEZ235 **(6B)**, sorafenib **(6C)**, and TSA **(6D)** were determined by MTT assays on proliferation of HGUE-C-1, HT-29 and SW620 cells. The average of at least four experiments is represented and referred as percentage of control. Error bars are the S.E.M.

One of the main objectives of the HGUE-C-1 cell culture generation was to develop predictive tests of chemoresistance in primary cultures obtained from biological samples of patients with colorectal cancer. HGUE-C-1 cells were treated with different drugs in order to test their sensitivity either when they were set in culture for the first time, or when they had been cultured for several passages, in order to determine its pharmacological profile. The results show that HGUE-C-1 cells are resistant to 5-FU and irinotecan compared to other colorectal cancer cell lines, as seen in Figures 4A and B. HGUE-C-1 cells also show resistance to cetuximab (Figure 5A), despite the patient was not treated with this EGFR antibody. Interestingly, HGUE-C-1 cells show sensitivity to erlotinib and gefitinib, inhibitors of the EGFR tyrosine quinase activity (Figure 5B). HGUE-C-1 cells are also sensitive to the m-TOR inhibitor rapamycin, the dual PI3K and m-TOR inhibitor NVP-BEZ235, the BRAF inhibitor sorafenib and the histone deacetylase inhibitor TSA (Figure 6A-D).

**Figure 7:**
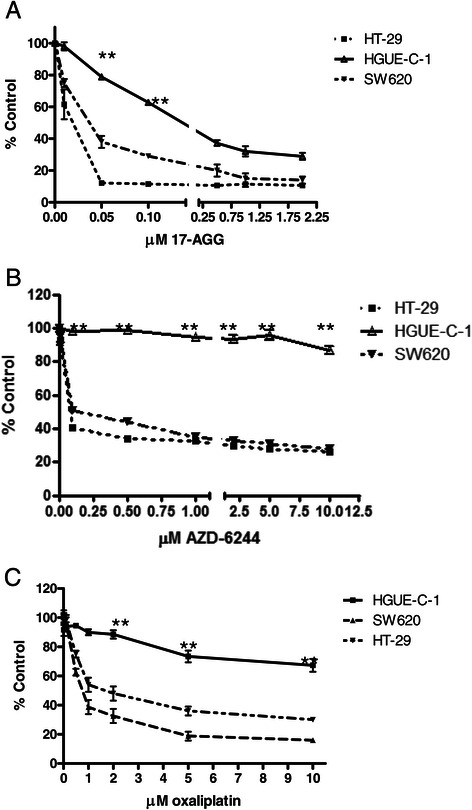
**Effects of drugs and chemotherapeutic agents on cell proliferation.** The effects of 17-AAG **(7A)**, AZD-6244 (selumetinib) **(7B)**, and oxaliplatin **(7C)** were determined by MTT assays on HGUE-C1, HT-29 and SW620 cell proliferation. The average of at least four experiments is represented and referred as percentage of control. Error bars are the S.E.M. *p < 0.05, **p < 0.01 versus HT-29 values.

Interestingly, HGUE-C-1 cells show partial response to the Hsp90 inhibitor 17-AAG (Figure 7A) and resistance to the MEK inhibitor AZD-6244 (selumetinib) (Figure 7B) and to oxaliplatin (Figure 7C), as compared to other colon carcinoma cell lines, despite the patient was not treated with these drugs.

### Drugs effects on HGUE-C-1 cell cycle

**Figure 8:**
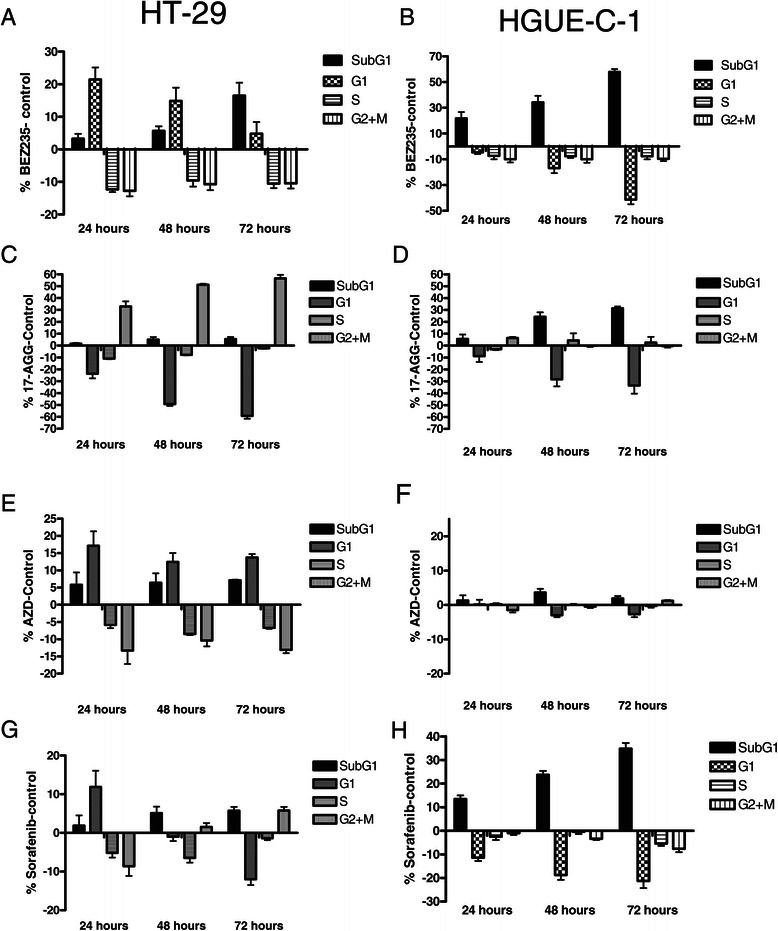
**Effects of drugs on cell cycle.** HGUE-C-1, HT-29 cells were grown, treated with NVP-BEZ235 **(8A, 8B)**, 17-AAG **(8C, 8D)**, AZD-6244 **(8E, 8F)**, and sorafenib **(8G, 8H)** for 24, 48 or 72 hours and cell cycle distribution of DNA content was determined by flow cytometry, and represented as the average of at least three separate experiments. HT-29 cells are shown in panels A,C,E and G. HGUE-C-1 cells are shown in panels B, D, F and H.

Taking in consideration that HGUE-C-1 cells show low levels of Rb protein expression, compromising the regulation of cell cycle checkpoints and, since most of the drugs tested play a role on cell cycle regulation, we analysed the effects of the different treatments on the distribution of DNA content of the different phases of the cell cycle by flow cytometry in HGUE-C-1 and HT-29 cells. Results in Figure 8(A-H) show that NVP-BEZ235, AZD-6244 and sorafenib (Figure 8A, E and G, respectively) are able to induce a G_1_ phase arrest in HT-29 cells, whereas 17-AGG induces a G_2_/M arrest, and subsequently accumulation in the SubG_1_ phase, indicative of apoptotic cell death (Figure 8C). However, HGUE-C-1 cells respond in a completely different manner after the same drug treatments. NVP-BEZ235 and sorafenib induce cell death without the G_1_ arrest observed in HT-29 cells (Figure 8B and H). Similarly but to a lesser extent, 17-AAG induces an increment in the SubG_1_ phase of the cell cycle (Figure 8D) in HGUE-C-1 cells, whereas this drug induces a G_2_/M arrest in HT-29 cells (Figure 8C). Almost no variation on cell cycle distribution was observed in HGUE-C-1 cells after AZD-6244, apart from a small and no significant increase in apoptotic cells without any evidence of cell cycle blockade (Figure 8F). Taking together, these results indicate that after treatment with these drugs, HGUE-C-1 cells undergo apoptosis with no previous arrest of the cell cycle. Similar commitment to apoptosis in HGUE-C-1 cells was observed after TSA treatment (data not shown).

### HGUE-C-1 identity control

**Figure 9:**
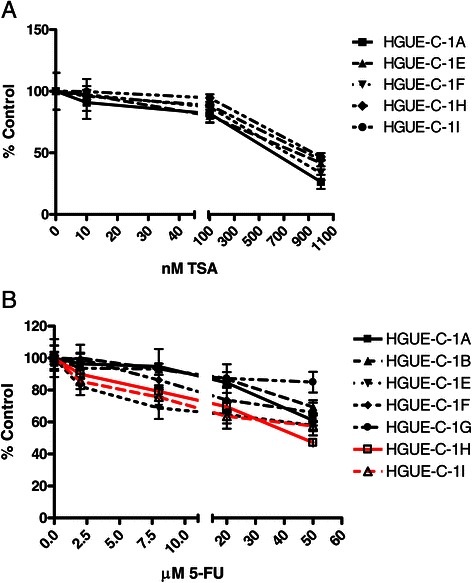
**Cell proliferation of clonal populations.** The effects of the histone deacetylase inhibitor TSA **(9A)** and 5-FU **(9B)** on cell proliferation of several clonal populations of HGUE-C1 cells was determined by MTT assays. The average of at least four experiments is represented and referred as percentage of control. Error bars are the S.E.M.

In order to identify putative molecular or genetic changes associated to cell culture and to validate the pharmacological profile shown in Figures 4, 5 and 6, different characteristics of HGUE-C-1 cells have been analysed in the initial primary cell cultures and in different cell passages. As a cross contamination control, we analysed the polymorphic microsatellite markers D2S123, D5S346 (previously mentioned) and D17S25in DNA samples obtained from two different passages. Both passages show the same pattern for the three MSI markers analysed, confirming a very high probability of identity (data not shown). Since HGUE-C-1 cells were obtained from ascites from a patient with colorectal cancer and not from the primary tumour excised two years before, and also because the patient had been heavily exposed to chemotherapy, we wanted to know the degree of heterogeneity inside the putative tumour cell population presented in the HGUE-C-1 cells. We isolated clonal cell populations from the HGUE-C-1 primary culture after 15 passages in culture (non-tumour cells have already undergone senescence at that point). We then analysed isolated clonal populations of HGUE-C-1 cells to determine MSI status (results shown in Figure 1) and their response to different drug treatments. We chose TSA because these cells are sensitive to this drug (Figure 6) and 5-FU because these cells have already been exposed to this chemotherapeutic agent. In Figure 9A we observed that the response to TSA treatment is quite similar in all clonal populations of HGUE-C-1 cells. However, different degree of resistance to 5-FU can be observed among the HGUE-C-1 clonal populations (Figure 9B).

Finally, we wanted to determine whether the results that we obtained testing the sensitivity to different drugs performed in HGUE-C-1 cells at the beginning of the culture correlate with the pharmacological profile of the cells after a well-established culture. We found a good correlation between the pharmacological profiles in both cases (data not shown). In addition and since HGUE-C-1 cells show cross resistance to different treatments, we studied the status of MDR1 and MRP1 protein levels and activity. We found that HGUE-C-1 cells do not show MDR1 and MRP1 expression and more importantly, they did not show any sign of activity of these ABC transporters, as determined by daunomycin uptake assays by flow cytometry (data not shown).

## Discussion

Herein we have characterized a new colon carcinoma cell line, HGUE-C-1, obtained from the ascitic fluid from a 76 years old patient with colon cancer. HGUE-C-1 cells represent an interesting model of study from different points of view. First, this cell line has been obtained from a tumour that belongs to a group of colon carcinomas lacking MSI, with loss of heterozygosity affecting the APC locus, and without *TP53*, *KRAS*, *BRAF* or *PIK3CA* mutations. These molecular characteristics are representative of less than 12% of all colon carcinomas [20]. Second, an extensive pharmacological profile of this cell line indicates that it represents a physiological model of chemoresistance (developed inside the patient) to 5-FU and irinotecan, drugs that have been used to treat this patient. Third, HGUE-C-1 cells show partial or total cross-resistance to drugs never used in the patient before, such as cetuximab, 17-AAG, oxaliplatin or AZD-6244. Finally, HGUE-C-1 cells are still sensitive to other drugs such as TSA, rapamycin, BEZ-235, sorafenib, erlotinib, gefitinib and others, suggesting that different pathways can still be targeted in patients with similar pharmacological profile and genetic background than the patient from which HGUE-C-1 cells were obtained.

As mentioned above, HGUE-C-1 cells are quite atypical when compared with other colon carcinoma cell lines, or when we analyse the usual genetic changes reported in colon carcinoma patients. In this sense, it is known that around 10-15% of colon carcinomas have MSI phenotype [21] and 50-55% of tumours carry mutations in *TP53* [22]. Similar percentages of mutations (40-50%) are found in *KRAS* and *BRAF* [23] and 17% of *PIK3CA* mutations have been determined in colon carcinoma patients [23,24]. This suggests that the probability that a patient will not carry at least one of the above mentioned genetic changes is quite low. For this reason, HGUE-C-1 cells constitute an interesting and unique cellular model of colorectal carcinoma.

Since HGUE-C-1 cells were not obtained from the primary tumour when it was resected two years earlier, it becomes difficult to determine the initial events in carcinogenesis in this cellular model. Besides the loss of heterozygosity affecting the APC locus that we have found in HGUE-C-1 cells, we have studied different signal transduction pathways looking for modifications that we could associate with HGUE-C-1 tumourigenesis and, we have compared these results with the findings obtained in HT-29 colon cancer cells, since this cell line constitutes a paradigm of the colon carcinogenesis pathway proposed by Vogelstein et al. [25]. HT-29 cells harbour *APC*, *BRAF*, *TP53* and *PIK3CA* mutations and MSI stability. HGUE-C-1 cells expressed very low levels of Rb protein compared with HT-29 cells. These low levels of protein expression correlate with low activity of Rb protein and with increased activity of E2F, as demonstrated by transient transfection of both cell lines with the appropriate reporter genes (Figure 2). Our analysis also shows that HGUE-C-1 cells overexpress p70S6K (RPS6KB1), which correlates with an increased activity of this kinase, as demonstrated by its high level of phosphorylation under serum starved conditions and also because under the same conditions, its substrate RPS6 appears phosphorylated. Analysis of other signalling molecules that belong to the Ras/Raf/Mek/Erk and PI3K/Akt/mTOR pathways also shows some differences between both cell lines. Interestingly, Erk1 and Erk2 appear constitutively activated under serum starved conditions in HGUE-C-1 cells, as demonstrated by the high levels of Erk 1/2 phosphorylation. In addition, no expression of HER3 was observed in HGUE-C-1 cells. The results obtained open the possibility of a putative role of RPS6KB1 or Erk 1/2 as second hits in HGUE-C-1 carcinogenesis. Interestingly, several publications suggest that RPS6KB1 may be involved in carcinogenesis. Ehrbrecht et al. [26] have analyzed 22 sporadic desmoplastic medulloblastomas by comparative genomic hybridization (CGH), finding in some of them an amplicon in chromosome 17 (17q22-24) where RPS6KB1 lies. Furthermore, they found significantly elevated transcript levels of RPS6KB1 as compared to normal cerebellum in 5 out of 6 desmoplastic medulloblastomas and in 4 out of 5 classic medulloblastomas analysed. The 17q23 amplicon has also been described by Sinclair et al. [27] in breast cancer and they even have suggested that RPS6KB1, TBX2 and PPM1D genes included in this amplicon may act as oncogenes. Genetic variation in RPS6KA1, RPS6KA2, RPS6KB1 and RPS6KB2 has been related to colon and rectal carcinoma in a recent article by Slattery et al. [28].

On the other hand, RPS6KB1 is regulated by growth factors such as EGF, PDGF and insulin, and is also regulated by mTOR [29], suggesting that it can be involved in cell growth regulation. In this sense, Sunayama et al. [30] have shown that inhibition of the PI3K/mTOR and the MEK/ERK pathways in glioblastoma stem-like cells induces an increase in the activity of the other pathways and that this cross-regulation disappeared when RPS6KB1 expression was inhibited by siRNAs. Using microarrays analysis, Chakraborty et al. [31] identified genes related to tumourigenesis of retinoblastoma. Their results suggested that loss or inactivation of Rb lead to dysregulation of the PI3K/Akt/mTOR pathway. To corroborate this hypothesis, the authors reported an increase in the expression levels of PIK3CA, Akt, mTOR and RPS6KB1 in retinoblastoma samples, when compared with normal tissue. Taken together, these data suggested that loss of APC function and over-expression of RPS6KB1 in HGUE-C-1 cells alone or in combination with low activity and expression of Rb protein, may constitute a driving force in the HGUE-C-1 tumourigenesis.

HGUE-C-1 cells constitute a quite interesting model from a pharmacological point of view since they can be used for the study of intrinsic chemoresistance to 5-FU and irinotecan, as shown in Figure 3. Since chemoresistance was naturally acquired by the patient after treatment with these chemotherapeutic agents, these cells represent a more physiological model than the usual chemoresistance cellular models obtained in the laboratory by selective pressure of parental sensitive cells treated with increasing concentrations of a particular drug. In addition, clonal populations of HGUE-C-1 cells show different degrees of resistance to 5-FU. We need to investigate whether they represent different stages of a unique molecular mechanism of resistance or else, an alternative molecular mechanism of resistance developed inside the patient. Other possibility is that due to tumour heterogeneity, clonal populations behave differently under the same conditions.

HGUE-C-1 cells were also treated in culture with cetuximab, an antibody against the extracellular domain of EGFR, since as mentioned above, this is a treatment used in advanced colon carcinoma patients and since HGUE-C-1 cells are wild type for KRAS and BRAF. As seen in Figure 5A, HGUE-C-1 cells show resistance to cetuximab despite the patient was not treated with this antibody. Interestingly, HGUE-C-1 cells show sensitivity to erlotinib and gefitinib, small organic molecules that inhibit the tyrosine kinase activity of the EGFR. This is an unexpected result since cetuximab, erlotinib and gefitinib act against the same target (EGFR). Mutations on exons 19 and 21 of EGFR have been related to tyrosine kinase inhibitor activity [32]. Since EGFR is expressed in HGUE-C-1 cells (Figure 3E), our results suggest that probably erlotinib and gefitinib act in HGUE-C-1 cells through the inhibition of a secondary target other than EGFR. Evidences showing inhibition of other tyrosine kinases by erlotinib and gefitinib have been previously published [33].

In addition, the effect of several drugs affecting different signal transduction pathways have been analysed on HGUE-C-1 cells. Our results indicate that HGUE-C-1 cells show similar degree of sensitivity to rapamycin, NVP-BEZ235, sorafenib and TSA than HT-29 cells. These results indicate that inhibitors of the PI3K/mTOR pathway may be a therapeutic alternative for colon carcinoma patients with resistance to 5-FU, irinotecan, oxaliplatin and cetuximab. HGUE-C-1 cells are also sensitive to TSA (Figure 6D) and SAHA (data not shown), indicating that epigenetic treatments may be also a therapeutical alternative for these patients.

HGUE-C-1 cell proliferation was also inhibited by sorafenib. We can postulate that the effects of sorafenib could be due to the inhibition of BRAF, since HGUE-C-1 cells show wild type BRAF. However, we cannot conclude that this is the case since sorafenib also induces inhibition of HT-29 cell proliferation, and this cell line harbours a mutation in *BRAF* (V600E) [34]. We postulate that probably, the inhibitory effect of sorafenib is mostly due to its capacity to inhibit other tyrosine kinases [35,36], as it occurs with erlotinib and gefitinib.

The Hsp90 inhibitor 17-AAG is able to inhibit HGUE-C-1 cell proliferation, however, significant higher doses of 17-AAG are needed to inhibit HGUE-C-1 cells than HT-29 cells (Figure 7A). This partial response to 17-AAG may be related to the resistance of HGUE-C-1 cells to cetuximab. In this sense, it is believed that Hsp90 inhibitors affect cell proliferation because they interfere in the relationship between Hsp90 and their clients [37]. Some of these clients are members of the HER family receptors [38]. The lack of cetuximab effect may indicate that EGFR does not play a significant role in HGUE-C-1 cells and, in this sense, 17-AAG effects on HER family members could be no significantly important for HGUE-C-1 cell proliferation.

HGUE-C-1 cells also show resistance to AZD-6244 (selumetinib), a Mek inhibitor (Figure 7B). The molecular mechanisms involved in this resistance remain unknown. However, a putative explanation arises analysing the effects of the different drugs on cell cycle distribution, as seen in Figure 8. When we compare the effects of sorafenib, NVP-BEZ235, 17-AAG, AZD-6244 and TSA, we observed that in the case of HT-29 cells, the different drugs induce a partial blockade in different phases of the cell cycle and later, some percentage of the cells undergo apoptosis, as determined by an increase in the subG_1_ population. However, this is quite different in HGUE-C-1 cells, since in this cell line, all drugs that affect cell proliferation induce directly an increase in the SubG_1_ phase, without previous accumulation in any of the phases of the cell cycle. Even in the case of AZD-6244 treatment, where a very low and no significant effect on cell proliferation was observed, we could detect a small increase in the subG_1_ cell population without any sign of cell accumulation in G_1_, as opposed to the results obtained in HT-29 cells. Taking together, our data suggest that defects on the G_1_/S checkpoint boundary in HGUE-C-1 cells due mainly to the low expression and activity of Rb, together with the increase on E2F activity (Figure 2C), are directly related to the lack of effect of AZD-6244 and to the partial effect of 17-AAG treatments in HGUE-C-1 cells.

Finally, HGUE-C-1 cells show resistance to oxaliplatin despite the patient was not treated with this compound. This resistance is important because as mentioned in the introduction, this compound increases survival in advanced colon carcinoma patients when added to the standard therapy with 5-FU or capecitabine and irinotecan [5-7]. The molecular mechanisms responsible for oxaliplatin resistance in HGUE-C-1 cells remain unknown. However, we believe that the low levels of Rb protein and activity may be involved in such mechanisms. Oxaliplatin damages the DNA inducing double strand breaks [39]. Since HGUE-C-1 cells do not show MSI and express wild type *TP53*, the expected response to oxaliplatin treatment in HGUE-C-1 cells should be p53 stabilization. Then, p53 will increase p21 and other proteins that will block the cell cycle in the G_1_ phase. Since as we have shown the G_1_/S checkpoint is seriously compromised by the low activity of Rb with the concomitant increase in E2F activity, HGUE-C-1 cells will be able to proliferate in such conditions. On the other hand, p53 will also increase the transcription of proapoptotic proteins such as NOXA and PUMA [40] but again, HGUE-C-1 cells are able to survive in this conditions probably because as mentioned before, loss of Rb or decrease in its activity have been related to deregulation of PI3K/Akt/mTOR signal transduction pathway [41]. This pathway has been extensively related with cell survival for example inhibiting the proapototic protein BAD [42]. In this sense, it is interesting to mention that p70S6K that is constitutively active in HGUE-C-1 is able to phosphorylate BAD [43].

## Conclusions

HGUE-C-1 is a new colon carcinoma cell line with interesting characteristics. First, it constitutes an example of a small percentage of colon carcinomas without MSI, with compromised APC activity but without *TP53*, *KRAS*, *BRAF* or *PIK3CA* mutations. Comparative analysis of different signal transduction pathways in HGUE-C-1 versus HT-29 colon carcinoma cell lines show that lack of APC activity, low expression and activity of Rb protein together with an over-expression and high activation of p70S6K and constitutive activation of Erk1/2, may be the driving force of HGUE-C-1 carcinogenesis. HGUE-C-1 cells also constitute an interesting cellular model to study chemoresistance acquisition to 5-FU, irinotecan, oxaliplatin, 17-AAG and AZD-6244. Interestingly, resistance to some of these drugs was acquired inside the patient as a consequence of the chemotherapeutical treatment (5-FU and irinotecan), being the partial or total resistance to 17-AAG, oxaliplatin and AZD-6244 unrelated to patients treatment. The molecular mechanisms regulating resistance to these compounds remain unknown. However, strong evidences suggest that the incapacity of HGUE-C-1 cells to be blocked in the G_1_/S cell cycle checkpoint can be the consequence of low Rb activity and the subsequent high E2F activity.

## Abbreviations

MSI: Microsatellite instability
APC: Adenomatous polyposis coli
Rb: Retinoblastoma
p70S6K: p70S6 quinase
TSA: Trichostatin
17-AAG: 17-allylamino-17-demethoxygeldanamycin
Hsp90: Heat shock protein 90

## References

[1] Jemal A, Bray F, Center MM, Ferlay J, Ward E, Forman D (2011). Global cancer statistics. CA Cancer J Clin.

[2] Andre T, Boni C, Mounedji-Boudiaf L, Navarro M, Tabernero J, Hickish T (2004). Oxaliplatin, fluorouracil, and leucovorin as adjuvant treatment for colon cancer. N Engl J Med.

[3] Twelves C, Wong A, Nowacki MP, Abt M, Burris H, Carrato A (2005). Capecitabine as adjuvant treatment for stage III colon cancer. N Engl J Med.

[4] Lin BR, Lai HS, Chang TC, Lee PH, Chang KJ, Liang JT (2011). Long-term survival results of surgery alone versus surgery plus UFT (Uracil and Tegafur)-based adjuvant therapy in patients with stage II colon cancer. J Gastrointest Surg.

[5] Haller DG, Tabernero J, Maroun J, de BF, Price T, Van CE et al. Capecitabine plus oxaliplatin compared with fluorouracil and folinic acid as adjuvant therapy for stage III colon cancer. J Clin Oncol. 2011;29: 1465–71. 10.1200/JCO.2010.33.6297 21383294

[6] Andre T, Boni C, Navarro M, Tabernero J, Hickish T, Topham C (2009). Improved overall survival with oxaliplatin, fluorouracil, and leucovorin as adjuvant treatment in stage II or III colon cancer in the MOSAIC trial. J Clin Oncol.

[7] Kuebler JP, Wieand HS, O’Connell MJ, Smith RE, Colangelo LH, Yothers G (2007). Oxaliplatin combined with weekly bolus fluorouracil and leucovorin as surgical adjuvant chemotherapy for stage II and III colon cancer: results from NSABP C-07. J Clin Oncol.

[8] Benson AB, Schrag D, Somerfield MR, Cohen AM, Figueredo AT, Flynn PJ (2004). American society of clinical oncology recommendations on adjuvant chemotherapy for stage II colon cancer. J Clin Oncol.

[9] Tournigand C, Andre T, Achille E, Lledo G, Flesh M, Mery-Mignard D (2004). FOLFIRI followed by FOLFOX6 or the reverse sequence in advanced colorectal cancer: a randomized GERCOR study. J Clin Oncol.

[10] Cassidy J, Tabernero J, Twelves C, Brunet R, Butts C, Conroy T (2004). XELOX (capecitabine plus oxaliplatin): active first-line therapy for patients with metastatic colorectal cancer. J Clin Oncol.

[11] Arkenau HT, Arnold D, Cassidy J, Diaz-Rubio E, Douillard JY, Hochster H (2008). Efficacy of oxaliplatin plus capecitabine or infusional fluorouracil/leucovorin in patients with metastatic colorectal cancer: a pooled analysis of randomized trials. J Clin Oncol.

[12] Moosmann N, von Weikersthal LF, Vehling-Kaiser U, Stauch M, Hass HG, Dietzfelbinger H (2011). Cetuximab plus capecitabine and irinotecan compared with cetuximab plus capecitabine and oxaliplatin as first-line treatment for patients with metastatic colorectal cancer: AIO KRK-0104–a randomized trial of the German AIO CRC study group. J Clin Oncol.

[13] Garcia-Alfonso P, Munoz-Martin A, Mendez-Urena M, Quiben-Pereira R, Gonzalez-Flores E, Perez-Manga G (2009). Capecitabine in combination with irinotecan (XELIRI), administered as a 2-weekly schedule, as first-line chemotherapy for patients with metastatic colorectal cancer: a phase II study of the Spanish GOTI group. Br J Cancer.

[14] Welch S, Spithoff K, Rumble RB, Maroun J (2010). Bevacizumab combined with chemotherapy for patients with advanced colorectal cancer: a systematic review. Ann Oncol.

[15] Dahabreh IJ, Terasawa T, Castaldi PJ, Trikalinos TA (2011). Systematic review: anti-epidermal growth factor receptor treatment effect modification by KRAS mutations in advanced colorectal cancer. Ann Intern Med.

[16] Xiao H, Yoon YS, Hong SM, Roh SA, Cho DH, Yu CS (2013). Poorly differentiated colorectal cancers: correlation of microsatellite instability with clinicopathologic features and survival. Am J Clin Pathol.

[17] Berg M, Guriby M, Nordgard O, Nedrebo BS, Ahlquist TC, Smaaland R (2013). Influence of microsatellite instability. Mol Med: KRAS and BRAF mutations on lymph node harvest in stage I-III colon cancers. Mol Med.

[18] Ogino S, Lochhead P, Giovannucci E, Meyerhardt JA, Fuchs CS, Chan AT. Discovery of colorectal cancer PIK3CA mutation as potential predictive biomarker: power and promise of molecular pathological epidemiology. Oncogene. 2014;33:2949-55. 10.1038/onc.2013.244 PMC3818472 23792451

[19] Zhu YF, Yu BH, Li DL, Ke HL, Guo XZ, Xiao XY (2012). PI3K expression and PIK3CA mutations are related to colorectal cancer metastases. World J Gastroenterol.

[20] Al-Sohaily S, Biankin A, Leong R, Kohonen-Corish M, Warusavitarne J (2012). Molecular pathways in colorectal cancer. J Gastroenterol Hepatol.

[21] Vilar E, Gruber SB (2010). Microsatellite instability in colorectal cancer-the stable evidence. Nat Rev Clin Oncol.

[22] Naccarati A, Polakova V, Pardini B, Vodickova L, Hemminki K, Kumar R (2012). Mutations and polymorphisms in TP53 gene–an overview on the role in colorectal cancer. Mutagenesis.

[23] Baldus SE, Schaefer KL, Engers R, Hartleb D, Stoecklein NH, Gabbert HE (2010). Prevalence and heterogeneity of KRAS, BRAF, and PIK3CA mutations in primary colorectal adenocarcinomas and their corresponding metastases. Clin Cancer Res.

[24] Kato S, Iida S, Higuchi T, Ishikawa T, Takagi Y, Yasuno M (2007). PIK3CA mutation is predictive of poor survival in patients with colorectal cancer. Int J Cancer.

[25] Fearon ER, Vogelstein B (1990). A genetic model for colorectal tumorigenesis. Cell.

[26] Ehrbrecht A, Muller U, Wolter M, Hoischen A, Koch A, Radlwimmer B (2006). Comprehensive genomic analysis of desmoplastic medulloblastomas: identification of novel amplified genes and separate evaluation of the different histological components. J Pathol.

[27] Sinclair CS, Rowley M, Naderi A, Couch FJ (2003). The 17q23 amplicon and breast cancer. Breast Cancer Res Treat.

[28] Slattery ML, Lundgreen A, Herrick JS, Wolff RK (2011). Genetic variation in RPS6KA1, RPS6KA2, RPS6KB1, RPS6KB2, and PDK1 and risk of colon or rectal cancer. Mutat Res.

[29] Heinonen H, Nieminen A, Saarela M, Kallioniemi A, Klefstrom J, Hautaniemi S (2008). Deciphering downstream gene targets of PI3K/mTOR/p70S6K pathway in breast cancer. BMC Genomics.

[30] Sunayama J, Matsuda K, Sato A, Tachibana K, Suzuki K, Narita Y (2010). Crosstalk between the PI3K/mTOR and MEK/ERK pathways involved in the maintenance of self-renewal and tumorigenicity of glioblastoma stem-like cells. Stem Cells.

[31] Chakraborty S, Khare S, Dorairaj SK, Prabhakaran VC, Prakash DR, Kumar A (2007). Identification of genes associated with tumorigenesis of retinoblastoma by microarray analysis. Genomics.

[32] Park SJ, Kim HT, Lee DH, Kim KP, Kim SW, Suh C (2012). Efficacy of epidermal growth factor receptor tyrosine kinase inhibitors for brain metastasis in non-small cell lung cancer patients harboring either exon 19 or 21 mutation. Lung Cancer.

[33] Carrasco-Garcia E, Saceda M, Grasso S, Rocamora-Reverte L, Conde M, Gomez-Martinez A (2011). Small tyrosine kinase inhibitors interrupt EGFR signaling by interacting with erbB3 and erbB4 in glioblastoma cell lines. Exp Cell Res.

[34] Wickenden JA, Jin H, Johnson M, Gillings AS, Newson C, Austin M (2008). Colorectal cancer cells with the BRAF(V600E) mutation are addicted to the ERK1/2 pathway for growth factor-independent survival and repression of BIM. Oncogene.

[35] Giovannetti E, Labots M, Dekker H, Galvani E, Lind JS, Sciarrillo R (2013). Molecular mechanisms and modulation of key pathways underlying the synergistic interaction of sorafenib with erlotinib in non-small-cell-lung cancer (NSCLC) cells. Curr Pharm Des.

[36] Jayanthan A, Bernoux D, Bose P, Riabowol K, Narendran A (2011). Multi-tyrosine kinase inhibitors in preclinical studies for pediatric CNS AT/RT: Evidence for synergy with Topoisomerase-I inhibition. Cancer Cell Int.

[37] Garon EB, Finn RS, Hamidi H, Dering J, Pitts S, Kamranpour N (2013). The HSP90 inhibitor NVP-AUY922 potently inhibits non-small cell lung cancer growth. Mol Cancer Ther.

[38] Baselga J. Treatment of HER2-overexpressing breast cancer. Ann Oncol. 2010;21 Suppl 7: vii36-vii40. 10.1093/annonc/mdq421 20943641

[39] Chiu SJ, Lee YJ, Hsu TS, Chen WS (2009). Oxaliplatin-induced gamma-H2AX activation via both p53-dependent and -independent pathways but is not associated with cell cycle arrest in human colorectal cancer cells. Chem Biol Interact.

[40] Li J, Lee B, Lee AS (2006). Endoplasmic reticulum stress-induced apoptosis: multiple pathways and activation of p53-up-regulated modulator of apoptosis (PUMA) and NOXA by p53. J Biol Chem.

[41] Gao N, Zhang Z, Jiang BH, Shi X (2003). Role of PI3K/AKT/mTOR signaling in the cell cycle progression of human prostate cancer. Biochem Biophys Res Commun.

[42] Spender LC, Inman GJ (2012). Phosphoinositide 3-kinase/AKT/mTORC1/2 signaling determines sensitivity of Burkitt’s lymphoma cells to BH3 mimetics. Mol Cancer Res.

[43] Harada H, Andersen JS, Mann M, Terada N, Korsmeyer SJ (2001). p70S6 kinase signals cell survival as well as growth, inactivating the pro-apoptotic molecule BAD. Proc Natl Acad Sci U S A.

